# Hurdles of Accessing HIV Treatment Among Homeless People Who Use Nyaope in Mogale City, Gauteng Province: An Exploratory Qualitative Study

**DOI:** 10.3390/healthcare13212807

**Published:** 2025-11-05

**Authors:** Betty Popi Ndlovu, Kebogile Elizabeth Mokwena, Mohora Feida Malebatja

**Affiliations:** Department of Public Health, School of Healthcare Sciences, Sefako Makgatho Health Sciences University, Pretoria Ga-Rankuwa 0208, South Africakebogile.mokwena@smu.ac.za (K.E.M.)

**Keywords:** adherence, barriers, hurdles, antiretroviral therapy, human immune deficiency virus, HIV treatment, nyaope use

## Abstract

**Background/Objectives**: The growing intersection between nyaope use and HIV infection constitutes a critical public health problem that undermines efforts to achieve universal access to HIV treatment in South Africa. Nyaope use is strongly associated with the increased risk of HIV of transmission. A significant amount of new HIV infections was linked to substance use through sharing of injectable needles. Despite significant progress made to increase public awareness and increase accessibility to HIV services, little is known about how addiction, stigma, and discrimination influence access to HIV treatment among homeless individuals who nyaope. This study explored the hurdles of accessing HIV treatment among people who use nyaope and are homeless in Mogale City, Gauteng Province. **Methods**: An exploratory descriptive qualitative research approach was employed among people who are homeless, living with HIV and using nyaope (PHHIVN) in Mogale City, between May and August 2024. Data were collected utilizing in- depth interviews in English, isiZulu and Setswana languages. Purposive sampling technique was followed to select participants, and a sample size of 25 participants was reached with a mean age of 32.28 and SD = ±5.54 years, of whom 21 (84%) were male, 3 (12%) were female and 1 (4%) identified as other. Audio recordings were transcribed, translated, and analyzed following inductive thematic analysis. **Results**: Social exclusion and fractured support system, prioritization of drug use, nyaope dependency, withdrawal symptoms, negative peer influence, socioeconomic factors and misconception about the interaction between nyaope and HIV treatment were reported as some of the main hurdles of accessing HIV treatment among PHHIVN in Mogale City, Gauteng Province. **Conclusions**: It is therefore concluded that access to HIV treatment among PHHIVN in Mogale City, Gauteng Province, remains a serious public health concern influenced by various hurdles. The development of tailored interventions to improve access and adherence to HIV treatment among this population group has potential to enhance the uptake of HIV treatment.

## 1. Introduction

Nyaope is a South African illicitly manufactured substance that contains several drug components such as heroin, cocaine, antiretroviral drugs, and marijuana [1,2,3,4,5,6,7]. Many researchers agree that its main ingredients include antiretroviral therapy, poison for rats, cannabis, heroin, detergent, pool cleaner, and battery acid [8,9,10]. The exact composition of nyaope is not standardized as it often varies across geographic locations [8,9,10]. In South Africa, where the study was conducted nyaope is also called “whoonga” [11,12], nyaope is usually smoked as a cigarette or injected into the blood stream by many people residing in various townships in South Africa [11,12].

Approximately, 20% of nyaope users have reported to be HIV positive in South Africa across various population groups [10,13,14]. Nyaope is known for its significant risk of dependency syndrome [13,15,16]. Some of the risky behaviours associated with the use of nyaope among PHHIVN include sharing of injectable needles, multiple intimate relationships, poly substance use, criminal behaviours, transactional unprotected sex and poor adherence to ART [17,18,19]. Its extensive availability in numerous South African communities facilitates habitual use among susceptible populations [16,20]. The combination of affordability, intense effects, and social determinants further increases the likelihood of dependence among users [12,13,16]. Nyaope’s addictive nature of nyaope contributes to elevated relapse rates [16,21,22], which are often linked not only to chemical dependence but also to psychosocial factors such as withdrawal symptoms, poor mental health, lack of support, and adverse environments [14,16,23]. These persistent relapses highlight the need for targeted interventions [15,16,21].

Despite global progress in addressing the HIV/AIDS pandemic, low- and middle-income countries continue to face major obstacles [1,2]. A major barrier is unequal access to HIV testing and antiretroviral therapy (ART), which is often influenced by national wealth, health infrastructure, and overall political will [3,4,5]. Although awareness and healthcare access have improved, many individuals still struggle to access treatment [1]. In 2022, about 9.2 million people living with HIV remained untreated, while approximately 5.5 million were unaware of their HIV status due to limited HIV testing services [6].

PHHIVN encounter multiple social and structural hurdles that limit their ability to access and continue with HIV treatment [13,24,25]. Social challenges encompass persistent stigma and discrimination from families, healthcare providers, and the broader community, which lead to feelings of shame and isolation [26]. Lack of family support and limited understanding of ART adherence further reduce engagement to care [10,27]. Structurally, challenges such as lack of a permanent physical address, concerns about the storage and security of medication while residing on the streets, and limited time to attend clinic appointments pose additional barriers to treatment [15,28]. Research also underscores that negative experiences in healthcare settings, including prolonged waiting times and unfriendly or judgmental staff attitudes, deter many substance users and people living with HIV from seeking care [14,15,16]. Collectively, these intertwined social and structural factors create a complex environment of exclusion and disengagement from health services.

Situations such as living on the streets or homeless are more common among PHHIVN exposing them to poor access to basic services, mental health conditions, poor nutrition and poor hygiene [24,29]. A study reveals that there is a correlation between homelessness and HIV amongst people using drugs [24]. Furthermore, mental health conditions such as depression act as barriers to ART initiation and adherence amongst people who use drugs [30,31]. Research has shown that addressing co-occurring mental health disorders can improve ART uptake and adherence among people who use drugs [30,32].

Significant correlations exist between nyaope usage and HIV status [10,13]. A considerable quantity of individuals living with HIV have been reported to use nyaope [12,13,15]. Poverty, low education, and poor access to healthcare contribute to both substance use and HIV vulnerability, particularly among disadvantaged youth [8,9,33]. These socioeconomic conditions reinforce a cycle of dependence and risk of infection. Incarceration further compounds these barriers, as people who use nyaope are frequently arrested for possession or related survival crimes such as theft and burglary [8,13,14,33,34]. Such experiences disrupt continuity of HIV treatment and rehabilitation services.

South Africa has taken steps to curb HIV spread and increase HIV treatment accessibility in recent years [35,36,37,38]. Starting from the year 2004, the South African Department of Health implemented availability of antiretroviral therapy free of charge at primary health care facilities nationwide [35,36,37,38]. While national HIV strategies have improved life expectancy and reduced HIV related morbidity [13,39,40,41], few interventions are specifically tailored to the needs of substance using populations. Current evidence suggests that the health system remains ill-equipped to provide specialized, integrated services for homeless individuals who use nyaope [42,43]. This study therefore aimed to explore the hurdles of accessing HIV treatment among PHHIVN in Mogale City, Gauteng Province, to better understand the personal, social, and structural barriers affecting their treatment engagement.

## 2. Materials and Methods

### 2.1. Study Area

This study was conducted at Non-Profit Organization (NPO) in Mogale City, Gauteng Province. The NPO provides HIV testing services and distribution of harm reduction packs to people who inject drugs, including those who use nyaope. The organization serves more than 100 clients living with HIV who use nyaope from various locations across Mogale City.

### 2.2. Study Design, Population, and Recruitment

An exploratory descriptive qualitative design was followed to explore the hurdles of accessing HIV treatment among PHHIVN in Mogale City, Gauteng Province. This is a suitable research design, that gives provision to investigate and explore a phenomenon of interest in depth such as the hurdles of accessing HIV treatment among homeless individuals who use nyaope. The target population for this study was PHHIVN in Mogale City, Gauteng Province. PHHIVN, above 18 years, who were addicted to nyaope over the past 6 months, who were willing to participate and give consent to participate were included in this study as participants. PHHIVN, above 18 years, who were addicted to nyaope over the past 6 months, but not willing to participate and give informed consent were excluded in this study. A sample size of 25 was reached, determined by data saturation. Data saturation is a stage where participants are no longer providing new information. Emerging codes were monitored from the data itself while checking with field notes to confirm data saturation. After the third round of fieldwork, no new information emerged. The researcher and supervisors evaluated the data and reached a consensus that data saturation was reached.

Permission to conduct the study was obtained from the NPO management and Sefako Makgtho Health Sciences University Research Committee (SMUREC/H/484/2023:PG). The researcher recruited potential participants in collaboration with the NPO. The researcher engaged with each participant individually, providing a briefing on the study. The purpose of the study was explained to each potential participant and those who were willing to participate, they were requested to go to the data collection room. Informed consent, both verbal and written, was obtained from participants prior to their involvement in the study, adhering to institutional and national ethical guidelines. Participants were duly informed that their involvement in the study was entirely voluntary, and they retained the right to terminate their participation at any point without incurring any repercussions concerning their eligibility for services provided by the NPO. To enhance privacy measures, all interviews were conducted within a confidential setting, specifically in a private room at the NPO. During the data collection and transcription phases, no names or identifiable details were documented. Instead, codes replaced personal identifiers, and access to the encrypted data files was restricted exclusively to the research team.

### 2.3. Data Collection

Data collection commenced post receiving clearance approval from SMUREC. Participants who agreed to form part of the study verbally were also requested to sign informed consent forms and identify themselves before any participants.

Data was collected through face-to-face in-depth interviews between May and August 2024. The study was guided by the Comprehensive Theory of Substance Abuse Prevention [44]. This theory provides a thorough explanation of substance misuse behaviours and advocates for individual level interventions that align with the root cause of the problem. An adapted interview guide was used to conduct in depth interviews, consisting of open-ended questions (Table S1). Each interview took approximately 60 to 90 min. The interviews were conducted in English, isiZulu, or Setswana depending on participants preference.

A digital translator was used to translate the data and verified by the transcriber. Private rooms were requested at the NPO to collect data, to ensure privacy and confidentiality for all potential participants including those that were identified at their “hotspot,” at Sivewright Street. Field notes were taken during the interviews and consolidated after each session to capture important details and researcher reflections. During data collection, participants were assured that their participation is voluntary, and they have the right to withdraw from the study at any given stage without any penalization. Interviews were conducted using the participants’ preferred language.

### 2.4. Sample Size and Sampling Technique

Purposive sampling technique was used to select participants who attend sessions at the NPO. The NPO provides services for PHHIVN from different age groups, ethnicity and genders with diverse backgrounds regardless of the duration of drug use and their HIV status. Attempts were made to achieve variety in the sample in terms of different genders, age, marital status, unemployment status, education level, years living with HIV and diagnose period. Purposive sampling technique was employed to select potential participants that represent similar characteristics. This sampling technique was best suited for this study, to identify a population category that has similar characteristics and to obtain detailed information and knowledge on the hurdles of accessing HIV treatment among PHHIVN in Mogale City, Gauteng Province.

### 2.5. Data Analysis

Qualitative data from audio recordings was used to generate transcripts. All 25 audio tapes recordings were first back translated from native languages to English, then transcribed into verbatim. The transcripts were imported to Nvivo 12 software for analysis. The authors independently read the transcripts thoroughly and repeatedly, to familiarize themselves with the data. Inductive thematic analysis approach was used to analyze data. Themes were generated from the data itself. The authors coded transcripts line by line to generate initial codes, which were then clustered into subcategories and broader themes. The supervisors and the independent co-coder analyzed the coded transcripts. The themes were presented in a meeting and where there were any discrepancies, they were discussed, and a consensus was reached. Coding discrepancies were discussed until consensus was achieved. No data was lost, all audio files, transcripts and notes were stored in password protected, encrypted folders accessible to the authors. Descriptive statistics were used to summarize participants’ demographic characteristics.

### 2.6. Trustworthiness

The researcher conducted the interviews. Before the participants left after the interviews, the information provided was confirmed with the notes to verify that the data obtained is a true reflection of participants’ intent. Themes were continuously refined through interactive comparison with the raw data to ensure they accurately reflected participants’ narratives. Credibility was ensured through recording of prolonged engagements with participants when conducting in-depth interviews. A thick description of the methodology and procedures was employed in this study, to allow application of the same methodology in a different settings and populations to ensure transferability. The supervisors kept an audit rail throughout the research process to ensure coherence of themes and used Nvivo software 14 for analysis.

## 3. Results

### 3.1. Socio-Demographics Characteristics of Participants

#### Sociodemographic Characteristics of PHHIVN

This study comprised of 25 participants. Their ages ranged from 20 to 49 years, with a mean age of 32.28 SD = ±5.54 years. Amongst the participants 21(84%) were males, 3(12%) were females and 1(4%) fell under the gender category of other. All 25 participants in the study were single. Most participants were unemployed at the time of the study, 24(96%), while a small proportion 1(4%) were employed. Nearly half of the participants 12(48%) had completed secondary education, a quarter had 6(24%) completed their matric, 3(12%) had tertiary education, 2(8%) had primary school education, and followed by another 2(8%) that had no formal education. With respect to HIV related data, 22(88%) of the participants were diagnosed with HIV after the use of nyaope, and 3(12%) were diagnosed with HIV before using nyaope (see Table 1 below).

### 3.2. Themes and Subthemes

#### Hurdles of Accessing HIV Treatment Among PHHIVN

This theme is about hurdles encountered by PHHIVN accessing HIV treatment. The participants revealed that social exclusion and fractured support system, nyaope dependence, withdrawal symptoms, peer pressure, socio-economic factors, misconception about the interaction between nyaope and HIV treatment were some of the hurdles faced by PHHIVN (refer to Table 2 below).

## 4. Discussion

The burden of HIV among PHHIVN remains a significant public health concern in many communities across South Africa. This study explored the hurdles of accessing HIV treatment among PHHIVN in Mogale City, Gauteng Province. The findings revealed six sub-themes as key barriers influencing access to HIV treatment: social exclusion and fractured support system, nyaope dependence, withdrawal symptoms, peer pressure, socio-economic factors, and misconception of the interaction between nyaope and HIV treatment.

Participants who took part in this study reported that PHHIVN are often subjected to stigma, moral judgement and abandonment by their families and communities, which affects their access to care. Similar studies also revealed perceived social exclusion as a major contributing factor to treatment discontinuation and a precursor to homelessness among PHHIVN [14,45,46]. In contrast, a study by [47], established that family support is positively correlated with enhanced adherence to HIV treatment and improved outcomes. Another study points out that substance use can cause moral stigma, resulting in exclusion from communal support structures [9]. Furthermore, individuals who use nyaope are often held in contempt within their communities, which not only amplifies shame but can also exacerbate further drug use as a coping mechanism [26]. Such experiences serve as a discouragement to seeking healthcare, particularly in public healthcare settings where families and community members are present.

Majority of the participants who took part in this study indicated that they spend most of their time and energy on finding money to buy nyaope rather than attending to their HIV treatment. This constant pursuit of funds is reflected in their dependency and their financial needs. Many participants reported that failure to obtain money for nyaope, result in avoidance to attend to their HIV treatment needs due to fear of withdrawal symptoms. As a result, their financial hardships serve as a dual barrier to their continued drug use while undermining adherence to their treatment. A similar study has identified financial constraints as a significant barrier to accessing HIV treatment [48]. Continuous engagement to HIV care is difficult when basic survival needs such as food, water, and shelter are unmet, underscoring the importance of integrating economic and social support within HIV programmes for PHHIVN.

Dependence on nyaope was found to be strongly linked to PHHIVN inability to maintain adherence to their HIV treatment. The participants disclosed that they have an uncontrollable craving for nyaope, which is essential for their daily functioning and emotional stability. Over prolonged use of nyaope, users develop dependency syndrome on the substance making it extremely difficult to cope without smoking nyaope on daily basis. This finding is supported by previous research that identified nyaope’s high addictive potential, which causes psychological and physiological dependence that is prioritized than their health needs [14]. Furthermore, ref. [14] it was also indicated that that the intense feelings experienced by PHHIVN when are out of nyaope causes feelings of helplessness and anxiety. The use of nyaope is addictive, hence PHHIVN find it difficult to cease smoking nyaope, an increase in smoking rate of nyaope leads to more desire.

The participants revealed withdrawal symptoms as a major barrier to accessing HIV treatment. These symptoms were commonly referred to as “cravings”, “alostros” or “down”, which include stomach cramps, sweating, loss of appetite, shivering, vomiting, dizziness, agitation, limited mobility, cold chills, diarrhoea and general body weakness. Such symptoms often intensified when participants attempted to reduce or delay nyaope use, which affect their endurance to long waiting times at the clinic. Similar findings were highlighted by previous research, that the physiological and psychological distress related to withdrawal symptoms can affect their treatment seeking behavior [16]. The severity and unpredictable nature of withdrawal symptoms act as a major deterrent to HIV treatment.

Peer influence emerged as a significant factor in shaping treatment behavior among PHHIVN said participants. Participants reported the difficulty of adhering to HIV treatment regimens in the presence of their peers. It was further reported by participants that instead of seeking their HIV treatment, they prefer to congregate with friends and smoke nyaope. According to a study by [16], peer influence frequently disrupts HIV treatment among homeless individuals who use nyaope. The study found that individuals using nyaope choose to smoke with peers rather than attend to their HIV treatment needs. Similarly, a study by [49], revealed that social groups that are characterized by similar practices of nyaope influences frequent use and relapse. Conversely, another study indicated that peer influence can often enhance cessation efforts and foster positive behaviour change [50]. Many PHHIVN prefer group settings to fulfil a sense of belonging, as they often face rejection by their families and communities. Influence from peers plays a huge role when it comes to adherence to HIV protocols. Therefore, support from peers can help improve HIV treatment adherence and harm reduction among PHHIVN by providing encouragement, motivation, and a safe space for them to continue with HIV treatment without fear of discrimination, stigma, or judgement.

Socio-economic factors such as unemployment, poverty, homelessness, know level of education, lack of shelter and food, and financial instability were revealed as some of the barriers to access HIV treatment among PHHIVN. Some of the participants indicated that their socioeconomic factors often force them to prioritize survival than to their HIV treatment. This aligns with prior studies indicating that people from low-income households often struggle to meet their basic needs, potentially prioritizing immediate survival over their healthcare requirements, including HIV treatment [51]. Homelessness further exacerbates these challenges by increasing the severity of HIV burden and diminishes access to treatment options [52]. Moreover, unemployment is associated with increased vulnerability and poor health outcomes due to limited access to stable income [53]. These socioeconomic conditions create an environment of instability and hinder adequate attendance to their HIV treatment.

PHHIVN, who took part in the study hold misconceptions that nyaope contains antiretroviral (ARV) components. The participants perceived that when consuming nyaope alongside prescribed HIV treatment could lead to harmful interactions. Contributing to treatment discontinuation, missed clinic appointment and reduced access to HIV services. Consequently, several participants deliberately discontinued their HIV treatment due to these perceived incompatibilities. This finding is consistent with the previous study indicating that misinformation and misconceptions surrounding drug interactions can significantly hinder adherence among people living with HIV [13]. Therefore, the misconception that smoking nyaope is equivalent to taking treatment, is attributed to lack of knowledge on HIV treatment composition and mechanism.

## 5. Conclusions

This study explored the hurdles of HIV treatment among PHHIVN in Mogale City, Gauteng Province, revealing multiple interrelated barriers to HIV treatment access including social exclusion and fractured support system, nyaope dependence, withdrawal symptoms, peer pressure, socio-economic factors, and misconception on the interaction between nyaope and HIV treatment The findings of this study highlight a critical gap in HIV and substance use education. The study identified a common misconception among PHHIVN holding a belief that smoking nyaope is equivalent to taking HIV treatment. Addressing this barrier requires targeted interventions integrated into harm reduction programmes and health care facilities. NPOs and community led programmes play an important role in peer education and substance use interventions. Strengthening collaboration between these services could further enhance HIV literary, eliminate misconceptions and improve HIV treatment. Furthermore, strengthening collaboration between healthcare providers, social workers, psychologists and nonprofit organizations is essential to improve adherence and retention to care among these individuals. For future research, longitudinal and mixed method studies are needed to examine the interventions that affect HIV treatment adherence and outcomes among nyaope users. Further chemical analyses could be beneficial in other provinces to help verify the persistence of ARV composition in nyaope.

## 6. Study Limitations and Strengths

The study was dominated by male gender and they young age group as participants. PHHIVN One strength of this study was choosing the NPO as the research setting. It provided easy access to participants who were directly involved in the topic, making it possible to gather rich and relevant data. However, the study was limited by the participants’ age and gender, as most respondents were males and fell within the age range of 20 to 39 years. This may affect how well the findings apply to other population groups across different geographical locations.

## Figures and Tables

**Table 1:** Socio-demographics information of participants.

Variable	Category	Frequency	Percentages
Age	20–29 years	9	36%
	30–39 years	13	52%
	40–49 years	3	12%
Gender	Male	21	84%
	Female	3	12%
	Other	1	4%
Marital status	Single	25	100%
Employment status	Employed	1	4%
	Unemployed	24	96%
Highest level of education	No formal education	2	8%
	Primary school	2	8%
	Secondary school	12	48%
	Grade 12	6	24%
	Tertiary	3	12%
Duration of nyaope use	0–5 years	7	28%
	6–10 years	7	28%
	11–15 years	9	36%
	16–20 years	1	4%
	21–25 years	1	4%
Years living with HIV	0–12 months	6	24%
	1–2 years	6	24%
	3–4 years	5	20%
	5–6 years	5	20%
	7–8 years	1	4%
	9+	2	8%
Diagnosed with HIV before or after using nyaope	Before using nyaope	3	12%
	After using nyaope	22	88%

**Table 2:** The findings of this study were summarized and demonstrated into a theme, sub-themes, illustrative quotes and interpretations of quotes below.

Theme	Sub-Theme	Quotes	Interpretation of Quotes
Hurdles of accessing HIV treatment among homeless individuals	Social exclusion and fractured support system	*“My family as well does no longer accept me as an individual because of drug use” (23-year-old female, participant 11).* *“The community is always negative on nyaope users, they see us as useless, and as thieves” (29-year-old male, participant 14).*	This sub-theme describes how PHHIVN experience social rejection and lack of support from their families and the community. Many participants reported being judged, abandoned and distanced by their relatives due to their drug use and its associated behabiours. Their relationships are further driven apart by acts of stealing and agression which b reinforces negative perceptions on the community. As a result, they feel isolated and stigmatised which intensifies their sense of exclusion and discourages tretment seeking behaviour.
	Prioritization of drug use	***“*** *My time is for smoking. I can’t wait a long time at the clinic. Secondly, you can see that we’re dirty, we aren’t the same as other people. I don’t have time. (26-year-old male, participant 8).* *“A person that doesn’t smoke nyaope has time for a lot of things, unlike us. For us as nyaope users to do something we must get that nyaope first. You will not do other things before smoking nyaope” (32-year-old male, participant 21).*	This subtheme refers to PHHIVNwhen collecting their treatment, including attending to clinic appointment. Some of the participants indicated that the financial struggles they experience contributes to this behavious, as they are frequently without food and money to go to healthcare facilities. Furthemore, many participants highlighted that their reliance on nyaope makes it difficult to prioritise anything else as they describe it as their primary enegry source. Homeless individuals daily strugges to obtainand use nyaope takes precedence over maintaining HIV treatment.
	Nyaope dependence	*“I need to smoke before anything. My day starts with al bag of nyaope and ends with a bag of nyaope” (32-year-old other, participant 1).* *“On a normal day I am unable to go to the clinic as nyaope controls my life” (27-year-old male, participant 25).* *“When we wake up the first thing, we do is smoke because there’s nothing you can do without smoking. That’s why in the end we don’t follow our treatment because maybe I didn’t have money to smoke in the morning. If I wake up without having it there’s nothing that I will do” (31-year-old male, participant 3).*	Nyaope dependence has been identified as a barrier to HIV treatment among homeless individuals who use nyaope. Many participants described that they are unable to function or begin their day without smoking nyaope. The intense urge and desire to continuously engage in nyaope lead to habitual and excessive use which create a cycle of dependency making it difficut to control usage. This constant need to feed the addiction overshadows other aspects of their lives.
	Withdrawal symptoms	*“It is “alostros”. It is the illness. That affects us, it’s like a stomach-ache, sweating. The stomach would feel like it’s getting tied up in knots. You can’t eat before you smoke. On the other hand, your bones and legs become locked” (35-year-old male, participant 2).* *“It’s heroine sickness. It’s called a ‘down’. I become weak, I become numb, dizzy, lazy to think, lazy work I become a numb person” (32-year-old male, participant 18).* *“There is this thing called ‘down’, that is the reason why a person has difficulties in getting treatment or anything that will better their life, you cannot do anything without smoking. Down is something in your mind. When you immediately think about nyaope that’s when you can’t do anything, you can’t even pick a box, you just drag yourself. Yoh! I feel weak, I don’t have strength. I don’t have energy and no appetite for food. Nothing can go in my body, until I smoke nyaope. You have a runny tummy, stomach-ache, such things”* *(34-year-old male, participant 10).*	Many participants referred to withdrawal symptoms as the clinical and psychological manifestations associated with nyaope use. Paricipants described these symptoms as severe stomach cramps, body weakness, dizziness, shivering, sweating, vomiting and agitation in the absence of nyaope in their bodies. These crinical symptoms often make it difficult for homeless individuals who use nyaope to cope, reinforcing continued use of nyaope as a relief of discomfort related to symptoms experienced.
	Peer influence	*“Our problem is that we have a certain mentality, we like to please each other. I would lie to you saying that tomorrow I’m going to fetch my treatment but the day I need to fetch my treatment, that doesn’t happen because I am with my friends” (31-year-old male, participant 3).* *“When I have my friends around me, I do not want to wake up in the morning and go get medication” (23-year-old female, participant 11).*	Some of the participants reported that their peers discourage them from adhering to their clinic appointment and adhering to their treatment. It must be noticed that homeless individuals who use nyaope use view their peers as their supportsystem, since many are not accepted or welcomed in their families and communities. As a result they find a sense of belonging and conform to their social groups and are obliged to comply with norms. Consequently, when adherence is not valued among the group, individuals are likely to adopt similar attitude.
	Socioeconomic factors	*“I am unemployed, I have no proper income to buy food so that I can take my treatment. Therefore, the little money I got; I will prefer to smoke than eat. If I have food, I will sell the food so that I can go smoke. It becomes difficult to take treatment while not eaten. When you are using nyaope, sometimes you have something that’s crucial for you, you forget about treatment. You focus on going to look for money so that you can smoke. This makes me to not take treatment as required” (36-year-old male, participant 23).* *“Sometimes living on the streets every day is something that causes us to default, or end up skipping days and not taking pills (32-year-old male, participant 21).* *“Because you live you on the streets that’s the thing that makes you not to have everything. On the streets you must always carry your things otherwise they get lost. Whether medication or what, when you’re on the streets you won’t focus. I’ve experienced that a lot. Now I’m living on the streets because of nyaope” (26-year-old male, participant 8).* *“You will fight in all ways just to get nyaope, any R20 you can get. You would be thinking about how you will get something to smoke and how you will get something to eat. You will end up not fetching your treatment anymore” (31-year-old male, participant 3).*	Poverty, homelessness and umplemployed were revealed as some of the hurdles of accessing HIV treatment among homeless individuals who use nyaope. These socioeconomic factors foster an environment of instability and prioritization of survival, making it difficult to focus on ones health. Many participants reported the lack of basic needs such as food, shelter and financial resources hinder their ability to take their treatment consistently, as HIV medication must be taken with food. Unemployment further deprives homeless individuals who use nyaope the ability to prioritise their HIV needs.
	Misconception about the interaction between nyaope and HIV treatment	*“Nyaope conceals or hides the virus. You won’t see that you have HIV. It is like medication to me. Because it hides the disease. You don’t feel the need to go to the clinic, you don’t feel as you would need your medication. When you smoke nyaope, it feels like you have taken your medication. Because you don’t feel anything, you feel healed” (39-year-old male, participant 16).* *Nyaope has got ARVs inside you know. So, when after using nyaope, it feels the same as I have taken my medication. I have other friend is a white guy and he’s been positive for one year and he hasn’t taken medication for one year and the sick has not progress to another level because nyaope has lots of ARVs inside” (32-year-old male, participant 1).* *“If you were sick now it makes you feel like you are no sicker. So, there is no need for treatment anymore. We are not going to go there the queue is long and we do not want to stay at the clinic long time. Most of the time if you are using nyaope, HIV hides behind nyaope. Because you can tell me I need to go to the clinic this time and you will find that at that time I am out to inject. I find it impossible to use nyaope and HIV treatment at the same time”(32-year-old male, participant 24).* *“I cannot smoke nyaope and take HIV treatment at once. The HIV treatment and nyaope do not have the same power, and other people’s system are weak, and others system are stronger, for instance, with me, I am able to smoke one bag(sachet) of nyaope a day until the following day. And when I must take my treatment; I first take my treatment and smoke afterwards, maybe I wait for about 1 h 30 min before I smoke so that the treatment can work within my system first” (35-year-old male, participant 18).*	Some of the participants reported that they believe there is an intyeractionbetween nyaope and HIV treatment. Some participants expressed that they avoid taking their HIV treatment because they believe it conflits with nyaope use. Participants percieved nyaope and HIV treatment as chemically incompatible, assumin that the usage of both simultaneously could be harmful. Other participants, believed that nyaope contains HIV treatment and therefore smoking nyaope serve as a substitute for HIV treatment. These misconceptions contribute to intentional not adherence to HIV tretment.

## Data Availability

The raw data supporting the conclusions of this article will be made available by the authors on request.

## Supplementary Materials

The following supporting information can be downloaded at: https://www.mdpi.com/article/10.3390/healthcare13212807/s1, Table S1: Interview guide with open-ended questions.

## Abbreviations

The following abbreviations are used in this manuscript:

HIV	Human Immunodeficiency Virus
ART	Antiretroviral Therapy
NPO	Non-Profit Organisation
PHHIVN	People who are Homeless, living with HIV and using Nyaope
