# Supplementary material for: Hurdles of Accessing HIV Treatment Among Homeless People Who Use Nyaope in Mogale City, Gauteng Province: An Exploratory Qualitative Study
Source: Healthcare (Basel). 2025 Nov 5;13(21):2807. doi: 10.3390/healthcare13212807 (PMC12607689; doi:10.3390/healthcare13212807)
Supplement: Supplementary file 1 [file healthcare-13-02807-s001.zip › healthcare-3855764-supplementary.pdf]

Table S1 Interview guide with open-ended questions

DATA COLLECTION TOOL

PARTICIPANTS INFORMATION

SECTION A: Socio-demographic questionnaire

1.	How old are you?		
2.	Sex	Male	
		Female	
		Other	
3.	Marital status	Married	
		Single	
		Widowed	
		Divorced	
4.	Employment status	Employed	
		Unemployed	
		Pensioner	
5.	What is your highest level of education?	No formal schooling	
		Primary school	
		Secondary school	
		Grade 12	
		Tertiary	
6.	How many years have you been living with HIV?		
7.	Were you diagnosed before or when using nyaope?	Before nyaope	
		When using nyaope	
8.	How do you use nyaope?	Smoke	
		Inject	
		Both smoke and inject	
9.	How long have you been using nyaope? In years		
10.	Does your partner also use nyaope?	Yes	
		No	
		I have no partner	
11.	Do you use “Bluetooth”	I often use blue-tooth	
		I do not use blue-tooth	
		I have used blue-tooth in the past	

		I have never used blue-tooth	
12.	My partner uses blue-tooth	Yes	
		No	
		I have no partner	
13.	Whom are you staying with?	Alone	
		With family	
		With friends	
		Homeless	
14.	How many times have you been in rehabilitation?		

SECTION B: Qualitative in-depth interview guide

**In-depth interview- Barriers of access to HIV treatment among nyaope users, in Mogale city.**
1. Can you tell me briefly how you knew about this centre?<br>a. Why did you come to the centre?<br>b. Who referred you or recommended that you come to the centre?
2. I understand that you are HIV positive, can you tell me how this centre assisted you and others to access HIV related services?<br>a. What specific services were you assisted to access?<br>b. Had you accessed such services in the past?<br>c. What exactly was done to assist you to access such services?
3. Now, can you tell me about your experiences of using nyaope?<br>a. When you started, did you know what it is?<br>b. Who introduced you to nyaope?<br>c. If you have seen people who use nyaope, what were your views about what it can do to a person?<br>d. Did you expect that you may end up being like some of them?
4. As a person who uses nyaope, are you treated differently or the same as someone who does not use nyaope when you go to health centres?<br>a. A. If so, give me examples of such differences?
5. As a person who uses nyaope, what are the things that make it difficult for you to access HIV treatment?<br>a. How do you manage such difficulties?<br>b. Are such difficulties common among other nyaope users who need HIV services?<br>c. How do these difficulties affect your willingness to access HIV related treatment?<br>d. What will make it easier for you and other nyaope users to access HIV related services?
6. How does the use of nyaope influence your treatment for HIV?<br>a. How does the clinic procedures affect your ability to access HIV treatment?<br>b. How does your life style influence treatment requirements for HIV?<br>c. How do health facility processes influence your intentions to access HIV treatment?

d. As a nyaope user, how does the community views influence your intentions to access HIV treatment?
7. What do you understand by adherence to HIV treatment protocol?<br>a. What is required for someone to be regarded as adhering to HIV treatment protocol?<br>b. What is needed to support someone to adhere to treatment protocol?<br>c. Considering your situation, to what extent do measure yourself regarding adherence to HIV treatment protocol?<br>d. What enables you to adherence<br>e. What frustrates your efforts to adhere?
8. Both nyaope use and HIV have some stigma attached to them, can you give examples of how stigma plays out in these two conditions?<br>a. Do you find it easier to deal with the stigma related to any of the two? Which one do you find easier to deal with?<br><br>HIV related stigma? Why?<br>Nyaope related stigma? Why?
9. What changes are needed to make it easier for people who use nyaope to access HIV related treatment?<br>a. What changes are needed by the centre<br>b. What changes are needed by the clinic?<br>c. In your opinion, what support do people who use nyaope need to assist them to access and adhere to HIV related treatment?
10. Is there anything you would like to share about how the use of nyaope affects HIV treatment?
